# A Review of Roof Harvested Rainwater in Australia

**DOI:** 10.1155/2018/6471324

**Published:** 2018-01-21

**Authors:** Chirhakarhula E. Chubaka, Harriet Whiley, John W. Edwards, Kirstin E. Ross

**Affiliations:** Environmental Health, Science and Engineering, Flinders University, GPO Box 2100, Adelaide, SA 5000, Australia

## Abstract

To address concern regarding water sustainability, the Australian Federal Government and many state governments have implemented regulatory mechanisms and incentives to support households to purchase and install rainwater harvesting systems. This has led to an increase in rainwater harvesting in regional and urban Australia. This review examines the implementation of the regulatory mechanisms across Australia. In addition, the literature investigating the potential health consequences of rainwater consumption in Australia was explored. Studies demonstrated that although trace metals such as arsenic, cadmium, chromium, lead, and iron were present in Australian rainwater, these metallic elements were generally found below the health limit guideline, except in high industrial areas. In addition, pathogenic or indicator microorganisms that include, but are not limited to,* Escherichia coli*, total and faecal coliforms,* Campylobacter*,* Salmonella*,* Legionella*,* Pseudomonas*,* Cryptosporidium*, Enterococci,* Giardia*,* Aeromonas*, and* Mycobacterium avium* Complex (MAC) have been detected in rainwater collected in Australia. However, epidemiological evidence suggests that drinking rainwater does not increase the risk of gastrointestinal disease. It was also identified that there is a need for further research investigating the potential for rainwater to be a source of infection for opportunistic pathogens.

## 1. Introduction

Australia is the driest inhabited continental land on earth [1, 2]. To mitigate drought effects on the sustainability of available water resources, many Australian states have introduced regulatory requirements and incentives for the installation of rainwater harvesting systems [3]. The primary intent of rainwater harvesting in Australia is to save on municipal water. However, in urban areas, with large impeded surfaces, rainwater harvesting is additionally used to manage surface runoff [4]. Rainwater, unlike municipal water, is rarely subject to multiple barriers that ensure its safety for human consumption [5]. In Australia, State Health Departments have produced guidelines suggesting that the public use municipal water for drinking and cooking [6, 7]. However, anecdotal evidence indicates that people are giving preference to drinking rainwater even when municipal water is available [8]. This review examines the factors that influence the use of roof harvested rain water in Australia and the potential human health consequences.

## 2. Methodology and Resources

This review has retrieved journal articles, published books chapters, and grey literature. PubMed and Scopus databases and Google Scholar were used to search resources from the Web. Google search engine (Google Inc. Mountain View, California, US) was used for grey literature search. Key words such as rainwater, contaminants, contamination, bacteria, microorganisms,* Escherichia coli (E. coli)*, faecal coliforms, trace metals, illness, gastroenteritis, outbreak, health guideline, health hazard, and Australia were used for database searches. The Flinders University Faculty Librarian Officer's services were used to recover archived and resources removed from the Internet. All documents included in the review were written in English, and no further translation was required.

**Figure 1:**
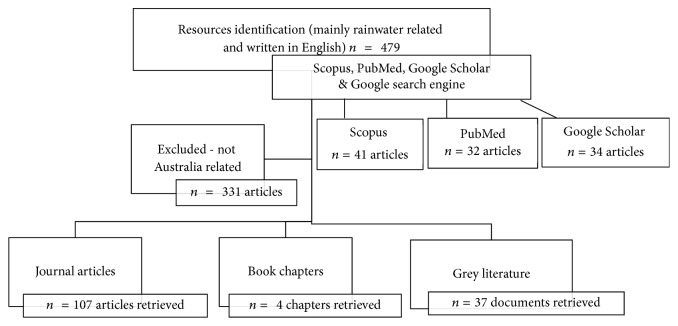
Methods of resources and materials search diagram.

Journal articles, books chapters, and grey literature resources searches were strictly limited to rainwater harvesting schemes and rainwater consumed in Australia. There were no exclusion criteria set for resources on pathogenic microorganisms and trace metals effects in humans. All resources that did not fall in that category were excluded even when found within the scope of rainwater harvesting. A total of 480 documents were searched, and 149 papers met the inclusion criteria (see Figure 1).

## 3. Water Sustainability

Climate change projections for Australia raise concerns over change in temperatures and rainfall patterns and ultimately over sustainable supply of water resources to communities [9]. In Australia, the average temperature has increased by 1°C from the middle of the 20th century [10]. The trends of rising temperatures over Australia are believed to have impact on groundwater renewal and aquifer recharges that support rivers perennial flow. The largest Australian perennial river, the River Murray, remains subject to drought conditions that prevail in its basin. A 2006 study indicated that, in periods of drought, 75% of water flowing in the Murray is used by riparian farmers [11]. Another challenge for Australia is the trend in population growth [12]. In December 2016, the Australia population was estimated to be 24.3 million people and the population growth was estimated to be 1.3% [13]. If current water policies remain unchanged, the demand in water resources is tipped to exceed water supply capacity in major Australian cities by 2025 [14].

## 4. Regulatory Framework Supporting Roof Harvested Rainwater

Historically, in periods of drought, rainwater provided drinking water to the first European settlers and to Indigenous Australians [15]. In rural and remote Australia, rainwater has provided drinking water to communities, and its use as source of drinking water is increasing in urban areas even though health authorities are reluctant to endorse rainwater as a safe source of drinking water [16]. In times of water shortages, rainwater is a useful substitute to municipal water. In 2017, the Royal Australian Air Force (RAAF) used harvested rainwater to supply water tanks to communities in Katherine (Northern Territory) after authorities found that municipal water was contaminated by per- and polyfluoroalkyl substances (PFAS) [17]. The PFAS is cumulative and nonbiodegradable chemical in human and at present, there is no strong clinical evidence that PFAS can cause cancer [18, 19]; however, links exist between human exposure to PFAS and testicular, kidney, and prostate cancer [20], the decrease in bones density, osteoporosis in women, and decrease in fecundity [21–24]. Before 1990, rainwater harvesting was not allowed in urban areas where municipal water was accessible [25]. However, over time rainwater harvesting and use became an accepted practice in urban areas [26].

Under regulation, building companies in many Australian states are now required to have rainwater tanks plumbed into new properties to comply with the Urban Development Industry (UDI). This is to save municipal water and to manage surface runoff [27]. In 2004, a regulatory framework was created to ensure that the Building Code of Australia (BCA) and the National Health and Medical Research Council (NHMRC) requirements for rainwater tanks installation were complied with. This was focused on water tank structures and water quality parameters (see Table 1) [28]. The regulatory framework, implemented by States and Territories, is managed by entities such as the National Water Initiative (NWI), the Australian Rainwater Industry Development group (ARID), the Master Plumbers and Mechanical Services Association of Australia (MPMSAA), and the National Water Commission Waterlines (NWCW) [28]. In New South Wales (NSW), the provision of Circular 14, 2002, of the state government requires that municipal water has connections that are separate to rainwater and that connecting pipes be labelled* “nonpotable water”* with a hazard identifier sign in place [29]. Subsequent to Circular 14, 2002, a policy on tanks plumbing was created by NSW Committee on Uniformity of Plumbing and Drainage Regulations (CUPDR) [30]. As a result, NSW Health Guidelines of January 2005 (GL2005-033) stated that well-maintained rainwater harvesting systems can provide a good source of water and suggested that adequate maintenance systems be in place when rainwater is used for potable [6]. Notwithstanding, New South Wales Health [3] warned the public on risks associated with drinking untreated rainwater. The warning message was echoed by Queensland Health and by Western Australia Health [31, 32]. A study on household drinking water attitudes found that many Adelaide residents were giving preference to drinking untreated rainwater when they had high quality municipal water supplied, and the preference was based on rainwater taste rather than on water quality [8]. A survey carried out with Currumbin residents (Gold Coast, Queensland) (*n* = 42) found that 100% of respondents used rainwater as source of drinking water and that 64% of households who consumed rainwater used basic sanitation practices that included water boiling, filtration, and ultraviolet treatment to improve rainwater quality [33]. In the Gold Coast region, high quality municipal water is supplied to communities by water utilities [34].

Based on the NSW Building Sustainability Index (BASIX) plans, an investigation was carried in 2011 on 52 tanks by Sydney Water [35]. The investigation found that Sydney families saved up to 38,000 L of municipal water in 2012, the equivalent of 21% of their annual water consumption [35]. In Canberra, a policy based on Australia Standards/New Zealand Standards 3500 (AS/NZS 3500) on tanks installation and plumbing and on rainwater use was enforced in 2010 [36]. In Queensland, Part 4.0 of the Queensland Building Sustainability (QBS) requires that Class 1 Building have a rainwater tank plumbed-in for nonpotable use [37]. Subsequent to Queensland Development Code, Mandatory Part 4–2 (QDC MP 4-2), a study carried by Umapathi et al., [38] to monitor municipal water savings in 20 households from mandated rainwater tanks, found that, in eleven months, families saved up to 36,1 kL of municipal water on an estimated 39.9 kL of rainwater annually consumed by households.

Subsequent to the Council of Australian Governments (CoAG) resolution of June 2003, Submission 158, the State Governments of South Australia, Victoria, New South Wales, and Queensland softened their attitudes and considered rainwater as a natural supply that is soft, clear, odourless, and good for drinking and cooking [29, 39]. In 2007, more than 1.5 million Australian families used rainwater as source of water, the equivalent of 19% of Australian households [27]. From 2007 onwards, the proportion of families that used rainwater as source of water steadily increased by 1% annually. In 2013, about 2.3 million families used rainwater as source of water, the equivalent of 26% of Australian households [40]. In both urban and regional Australia, an estimate of 50% of rainwater harvesting systems identified was plumbed in for indoor use [41]. In 2013-2014, about 46 GL of rainwater was consumed by Queensland households compared to 40 GL in New South Wales, 20 GL in South Australia, 10 GL in Western Australia, 0.9 in Canberra, and 0.5 GL in Northern Territory [42].

## 5. Incentives to Rainwater Harvesting

Under the Water for Future Initiative (WFI), the Australian Federal Government introduced a rebate scheme in 2009 to help families purchase and install new rainwater harvesting systems for nonpotable purpose [49]. A total of 14,625 rebates, the equivalent of $7 million, were offered to families by the Federal Government. The program ended in June 2011 [49]. Out of 14,625 rebates led by the Federal Government, 55% of rebates were offered to families in New South Wales with 18.2% offered to families in South Australia, 13.7% offered to families in Victoria, and 0.3% offered to families in Tasmania [49]. The government rebates were offered in terms of discount on tank purchase and the money was paid to tanks suppliers or to builders. No rebate was offered to families in Northern Territory. In addition to the Federal Government rebates, many Australian State governments have developed regulatory mechanisms to promote rainwater harvesting plans (see Table 2).

Because of the rebates policy, 32% of families with houses that met the standard requirements installed rainwater harvesting systems [40]. The number of new installed tanks increased in capital cities more than in regional Australia. Hence, 47% of Adelaide households installed new tanks followed by Brisbane households (44%) and Melbourne [37, 40]. Likewise, 86% of Hobart households plumbed in their tanks for nonpotable use followed by Melbourne household (23%) [75]. The reason to install new tanks differed from households. It was reported that 60% of Melbourne households installed tanks to save municipal water, 38% installed tanks to comply with water restrictions measures, and 24% installed tanks to save on water bills [75].

## 6. Contamination of Rainwater and Quality Assessment

As with surface water, rainwater can be contaminated with coarse and fine particulate matters, chemicals, microorganisms, metals, and ionic elements, which may be detrimental health effects [76, 77]. Previous studies have suggested that the human health consequences associated with rainwater are low in intensity and are linked to the type of rainwater harvesting systems design and maintenance [64, 78]. It has also been suggested that many people can develop immunity to rainwater pathogens or that they suffer from asymptomatic infections of minor infection with mild symptoms that go unnoticed [79].

## 7. Trace Metals in Rainwater Stream

There are twenty-three metals known to be toxic to human [80]. Out of these metals, arsenic, cadmium, cobalt, chromium, copper, mercury, manganese, nickel, lead, tin, uranium, and titanium are classified as highly toxic [78]. Trace metals occur in many environmental matrices [81] and naturally in earth crust [80]. These metals are believed to spread in the environment from metal smelters and wastes processing plants [78] or from mining and industrial discharge, air pollution fallout, urban runoff and sewage effluent, and traffic emissions [82]. In rainwater, contamination with trace metals may come from the catchment and storage structures [83, 84] or can be carried and deposited on the roof by the wind and washed into the stored rainwater [85]. A study that involved the survey of 34 tanks in subtropical Australia (Queensland) found that 65% of ionic contaminants and trace metals detected in rainwater were collected in the atmosphere by water during rainfall events, with the remaining 35% linked with corrosion on structure materials, paints, and lead flushing [60]. Case studies have also indicated that rainwater with a pH lower than 6.5 can be corrosive on structures and dissolve metals and leach them in stored rainwater [78]. In Australia, studies on rainwater contamination by metals are still limited in scope. In the studies that have been done, often samples were found positive to metals but generally within accepted health guidelines [57]. However, in former industrial corridors and raw material export terminals, studies found metals above health limits in rainwater samples (see Table 3).

In Newcastle (NSW), a study found seasonal variations in trace metals load in rainwater [56] (see Table 4). In the summer months, lead was detected 1,050 times above health limit with zinc detected 241 times, manganese 164.6 times higher, nickel 136 times higher, cadmium 85 times higher, and arsenic detected 55 times higher. The study did not determine the origin of these metals; however, the detection of lead and manganese in higher proportions in Newcastle rainwater samples would have links with high industrial activity. Until the 1950s, silicate manganese ore (MnSiO_3_) was mined in New England and processed in Newcastle by Broken Hill Proprietary Company Ltd. Steelworks (BHP Steelworks Ltd.) to make alloys [86]. It should be noted that, in many ore deposits, silicate manganese occurs with lead, nickel, zinc, and copper [87].

Years after the mine closure and with time and weathering, the mine remaining overburden breaks down and during summer months, the drier conditions enable dust bearing manganese to be released into the environment. Similarly, the excess amount of lead found in Newcastle rainwater samples may be from the same source as BHP Steelworks Ltd. used coal as fuel [86]. It has been reported that lead occurs at low level with black coal mined in the Hunter Valley adjacent to Newcastle [88]. In addition, Newcastle is Australia largest terminal coal export [89]. Like in Newcastle, a study conducted in Brisbane (Queensland) found that lead, cadmium, and iron were generally detected above accepted health limits in rainwater samples collected in drier months [90].

Three studies conducted in Melbourne found that lead was a major contaminant of rainwater [53]. Study 1 involved the analysis of water samples from 6 small tanks collected from glazed tile rooftops of 0.1 m^3^ storage capacity each. Study 2 involved 9 normal sized tanks and Study 3 investigated 40 tanks. It was reported that Study 1 detected lead 50 times above health standards. Out of 40 tanks investigated in Study 3, samples from 11 tanks contained lead above health limit. Lead flushing along with roof structure and tanks materials were believed to be source of rainwater lead content. Study 2 recorded a pH of between 4.3 and 4.9, making rainwater acidic and eventually corrosive on structures.

A study which investigated dust impact from the Port Adelaide Waterfront Redevelopment Project (PAWRP) at Lefevre Primary School in Adelaide detected antimony, arsenic, barium, cadmium, chromium, lead, and manganese in relatively high concentrations [91]. Samples were collected in Classroom 13 (Site A) and in the Gymnasium (Site B). It should be noted that metals found in the classrooms could also be found in dust on building rooftops and in event of rainfall, they would make their ways in stored rainwater should buildings in the area be fitted with rainwater harvesting systems as noted by Gikas and Tsihrintzis [84]. The source of these metals was not otherwise identified. However, Lefevre Primary School is located at the edge of Port Adelaide former industrial precinct. In the area, General Motors Holden (GMH) operated a Car Assembly Plant (CAP) in Birkenhead waterfront, few meters away from the school location before it moved to Woodville in 1923 [92]. Thus, metals found at Lefevre Primary School might have been sourced by dust blown from the former industrial precinct, given its proximity with the school.

## 8. Potential for Human Exposure

Metals in human have limited beneficial effects [82]. At high intake, hexavalent chromium (chromium VI or Cr^+6^), arsenic, cadmium, mercury, lead, and barium are toxic metals [82]. At lower intake, copper, cobalt, trivalent chromium (chromium III or Cr^3^), and nickel are essential nutrients in human [78, 93]. While trivalent chromium is an essential nutrient for sugar balance and fat metabolism in human, long-term exposure to hexavalent chromium is poisonous [81, 94]. Cadmium is a cumulative toxin which affects kidneys, deforms human reproductive and endocrine systems, and disturbs bones metabolism [95]. Lead is as noxious as cadmium and hexavalent chromium. Studies have found that lead contamination can trigger mental and personality disorder in children until late puberty [96, 97]. In adults, long-term exposure to lead can cause anaemia and damage the human Intelligence Quotient (IQ) and the reproductive organs in males [96]. In pregnant women, longer exposure to lead can trigger miscarriage [96]. At high intake, arsenic can impair the human cardiologic system and damage the liver and the central nervous system [93]. In pregnant women, lead can freely pass from the mother to the child and trigger lead prenatal contamination [98].

The review identified no incident of illness caused by drinking rainwater contaminated by trace metals. However, the lack of evidence could not conclude the absence of disease linked with drinking rainwater contaminated by metals in the community, given the number of Australians who are using rainwater as source of drinking water. Incidents of illness may exist in the community but may not be reported to health authorities. Metal poisoning side effects are cumulative in scope and it takes time for the symptoms to appear, making incidents of metals poisoning hard to diagnose in a timely manner [99]. Nevertheless, studies indicate that incidents of illness caused by metals poisoning through other routes are recorded in the community [100–102].

## 9. Microbiological Contamination

The likelihood of rainwater to contain microorganisms is high [64]. Generally, microorganisms found in rainwater are assumed to be from birds and small mammals that live around suburban areas. This is supported by a study carried by Ahmed et al. [103] on 22 rainwater tanks in Brisbane and in the Gold Coast region, where suburban birds and possums were found to be the vectors of all* E. coli* strains that were isolated from rainwater. Likewise, faecal matter that contains these microorganisms can also be carried with the dust and windstorms and be deposited on catchment areas and get discharged into harvested rainwater [104]. In underground tanks, faeces of large animals and humans collected by surface runoff can enter improperly designed, damaged, or unsealed tanks [105]. This review found no study carried out on underground tanks in Australia.

Table 5 shows that microorganisms such as* E. coli*, total and faecal coliforms,* Campylobacter, Salmonella, Legionella, Pseudomonas, Cryptosporidium*, Enterococci,* Giardia*,* Aeromonas,* and* Mycobacterium avium* Complex (MAC) have been detected in rainwater harvested in Australia. Commonly detected bacteria are* E. coli* and Enterococci. Considering the degree of* Enterococcus *spp. virulence and their observed level of prevalence in rainwater, the bacterium is also used as faecal indicator organism in the determination of rainwater microbiological quality, in addition to traditional* E. coli* [106, 107]. In an earlier study, Ashbolt et al. [108] argued for the need to use Enterococci in the determination of recreational water quality. In line with this proposal, it was suggested that rainwater be subject to reasonable sanitation works, if rainwater is to serve as source of drinking water [109].

Case studies have shown that rainwater harvested in many locations of Australia is generally of poor microbiological quality [9]. A study that involved the quantification of microorganisms of faecal origin in rainwater harvested in Queensland detected* E. coli* in the range of <1 to 3060 ± 456 CFU 100 mL, whereas Enterococci and* C. perfringens* were detected in the range of <1 to 3400 ± 700 CFU 100 mL [110]. In southeast Queensland, study on the assessment of health risks linked with rainwater used for potable and nonpotable purpose found that 10.7% of samples contained* Salmonella* and 9.8% of samples were found positive for* Giardia lamblia*, 5.6% positive for* Legionella,* and 0.4% positive for* Campylobacter jejuni* mapA genes [111]. The study tested 214 samples collected from 84 tanks.

While epidemiological evidence links* E. coli* and incidents of gastroenteritis illness [112, 113], studies have shown that not all strains of* E. coli *are pathogenic, although some can cause gastroenteritis, haemorrhagic colitis, and kidney failure, which can be fatal [114, 115]. Enterococci typically cause a gastrointestinal illness but can also cause urinary tract and blood infections [116]. A study carried on rainwater microbial content has found that, in Australia, 60% of tanks surveyed contained* E. coli* [61]. Another study conducted in Queensland detected* E. coli* in 15 tanks over 35 tanks and Enterococci in 21 tanks over 35 tanks. The rate of prevalence was 48.5% for of* E. coli* and 60% for Enterococci [66].

A survey of 72 rainwater tanks in Brisbane and Gold Coast (Queensland) detected* E. coli* and Enterococci in 74% and 94% of tanks, respectively. Another study carried in 2015 in Brisbane on rainwater detected* E. coli* and Enterococci in similar proportions [117]. The colony-forming unit of organisms count (CFU/100 mL) ranged from 0.3/100 mL organisms to 3.7/100 mL organisms [117]. In water,* E. coli* can survive between 15°C and 18°C for 3 months [118]. In harsh environment,* E. coli* lifespan can sharply vary from some days to few hours [118]. It should be noted that, in water,* Enterobacteriacae *bacteria have very similar lifespan to* E. coli* [119].

In the early 1900s, total coliforms and* E. coli* were believed to naturally occur with faeces and the detection of total coliforms implied the presence of* E. coli* [118]. Gradually, the detection of total coliforms in the absence of faeces became evident [120]. Since total coliforms can grow in the environment without reference to faeces, the bacteria are no longer surrogate indicator of water faecal contamination. The bacteria have since been replaced by* E. coli* and Enterococci [121]. In Australia, the water quality standard for potable water is 0/100* E. coli* CFU/mL [122]. The guideline extends to faecal coliforms and these bacteria, like* E. coli*. It is recommended that all strains of faecal coliforms be 0/100 CFU/mL for all points in the drinking water treatment and supply chain [123].

*Campylobacter* and* Salmonella* are typically considered foodborne illness causative agents, but other environmental sources including water can play a role in disease transmission [124, 125].* Campylobacter* is the causative agent of campylobacteriosis and the leading cause of gastrointestinal illness in Australia [126, 127].* Salmonella* is the causative agent of salmonellosis gastroenteritis which has been increasing in incidence in Australia over the last decade [128, 129]. A study conducted in southern Queensland detected* Campylobacter* spp. and* Salmonella* spp. in 7 tanks over 35 tanks tested [66].* Campylobacter* is shed in the faeces of infected humans and animals and the bacteria cannot replicate outside a host [130]. However,* Campylobacter* has been shown to survive between 29 and 120 days in environmental water sources [131, 132], and* Salmonella *can replicate outside a host and have been shown to survive in water source with minimal carbon content for at least 63 days [133].

*Legionella* and MAC are opportunistic pathogens [134].* Legionella* is the causative agent of Legionnaire's Disease, an atypical pneumonia infection and Pontiac fever, a mild febrile illness [135]. MAC can cause a range of infections including musculoskeletal infections, respiratory disease, lymphadenitis, and skin and soft tissue infections [136].* Legionella* can easily grow in potable water distribution systems, in freshwaters and thermal waters, and in compost and potting mix, and the bacteria optimal living temperature is in the range of 20 and 45 degrees Celsius [135]. Likewise, MAC are ubiquitous in the environment and can grow in soil and water sources including potable water distribution system [137]. When MAC are exposed to harsh environmental conditions, the bacteria enter dormancy lifecycle and its lifespan can become longer [138].

## 10. Epidemiological Evidence

Since incidents of illness caused by drinking untreated rainwater may be limited to small numbers of people, it is difficult to identify individuals with infections linked with drinking rainwater in the community by means of epidemiological tools [139]. As such, real incidence of infections linked with drinking rainwater may be underreported or simply not reported [140]. A quantitative microbial risk assessment carried in Queensland by Ahmed et al. [140] reported that over 1,000 people who annually drink rainwater daily, the chance to develop an infection was estimated for* Giardia lamblia* to 44–250 individuals and for* Salmonella *spp. to 85–520 individuals. Irrespective to the findings, the assessment concluded that, in Queensland, risks of infections linked to drinking untreated rainwater were exaggerated.

In Australia, there are limited epidemiological studies on rainwater consumption and incidents of gastroenteritis [141]. A study conducted by Rodrigo et al. [50] on 300 families that used rainwater as source of drinking water found that rainwater consumption did not extensively contribute to gastroenteritis incidents. Later, Rodrigo et al. [52] and Hamilton et al. [142] highlighted the lack of strong epidemiological evidence that links gastroenteritis and rainwater consumption, albeit case control has indicated a relationship between drinking untreated rainwater and the illness. A study by Heyworth et al. [143] indicated that, in South Australia, children who drank rainwater were not found to have a higher level of gastroenteritis incidents, compared to their peers who drank centralised municipal water. The observation was later supported by Dean and Hunter [144] and Abbott and Caughley [145] argued that, in South Australia, 42% of households drink untreated rainwater with limited gastroenteritis risks.

While the emphasis in the study by Hamilton and Ahmed was on* Legionella* and* Mycobacterium avium* complex (MAC), Hamilton et al. [146] indicated that drinking untreated rainwater would cause a cervical lymphadenitis in children and lead to disseminated infections in immune compromised adults. These authors suggested that rainwater be limited to car and clothes washing. The review has identified three incidents of disease outbreaks linked with drinking untreated rainwater in Australia. The first outbreak caused by* Campylobacter* was identified in Queensland [68], with a second linked to* Salmonella* identified in Victoria [74], and a third caused by* Giardia lamblia* identified in New South Wales [72] (see Table 6). Incidents of illness were recorded in aged care facilities and holiday camps [71].

## 11. Conclusion

To mitigate growing concerns over the sustainability of water resources supply to communities, the Australian Federal Government and many state governments have developed regulatory mechanisms and incentives to support families purchase and install rainwater harvesting systems to supplement municipal water. Increasingly, rainwater harvesting has become more common in Australian capital cities and in regional Australia. Guidelines on rainwater harvesting and use and on tanks installation are in place in most Australian states and territories. A mandatory rainwater tank plumbing policy on houses in new developments is enforced in South Australia, Victoria, New South Wales, and Queensland and having a rainwater tank on large extensions has become mandatory. Many Australians are using untreated rainwater as source of drinking water.

In comparison with municipal water supplied to communities, rainwater harvested in Australia can be of poor quality. Contamination with trace metals is generally low, except in some locations with large industry pollution. Contamination with microorganisms is common, but there is limited epidemiological evidence to suggest that exposure to gastrointestinal pathogens in rainwater results in an increase likelihood of gastrointestinal illness. However, there is a need for more research investigating the risk posed by opportunistic pathogens, particularly in susceptible populations. Notwithstanding increasing support to the industry, the Australian Federal Government and all States Health Departments recommend the public to exclusively limit rainwater use for nonpotable purposes to avoid risks of contamination.

## Figures and Tables

**Table 1:** Regulations and specifications for rainwater tank installation.

States	Regulation	Specification	Water use	Reference
South Australia (SA)	Development Act 1993 and Development Regulations 2008 which complete the Waterworks Act 1932 and the Environment Protection Act 1993 completed by the Waterworks Regulations 1996, the Public and Environmental Health Act 1987, and the Natural Resources Management Act 2004.	Houses in new developments and house extensions greater than 50 m^2^ must have an additional water supply to supplement municipal water. SA Water regulates the tank plumbing policy in fulfilment of the Waterworks Act 1932 and Waterworks Regulations 1996.	In new Class 1 buildings, all tanks must be plumbed into the house and water used for toilets flushing, hot water systems, or cold water outlets in the laundry.	[43]

Victoria (VIC)	The 5-star standard for all new houses in Victoria (Victoria Building Code 2005) of 1st of July 2005 requires that new houses have a rainwater tank plumbed to the house. Regulations 2008, Version 013, SR number 136/2008, in fulfilment of Part 12A of the Building Act 1993.	New Class 1 buildings are required to have rainwater tank of not less than 2,000 L plumbed for toilet flushing. The roof area shall be greater than 50 m^2^ to meet the tank plumbing requirements and maximize the harvest.	All tanks must be plumbed for toilets flushing. Rainwater must be separated from municipal water supply and the overflow directed into storm water drainage system.	[44]

New South Wales (NSW)	The New South Wales Health Guidelines of 2005 (GL2005_033 of January 2005) which complete the Building Sustainability Index (BSI) require that new residential houses in NSW use less municipal water.	The installation of tanks of capacity greater than 10,000 L requires Sydney Water approval to avoid infringing on Sydney Water structure or easement.		[45, 46]

Queensland (QLD)	The Queensland Building Regulation 2006 (QBR 2006), Subordinate Legislation 2006 number 227 under Division 2. The QDC-MP 4-2 of 2007 regulates rainwater tank installation.	The QDC-MP 4-2 in place from 2007 recommends that new houses from 100 m^2^ roof area to have rainwater tank of 5 kL installed by builders at a cost of $4,000 paid by home owners.	Tank plumbed in for toilet flushing, clothes washing, and an external tap to save municipal water use up to 70 kL annually and 42 kL for detached houses.	[31, 47]

Western Australia (WA)	No governing policy in place.	The health department advises the public to limit rainwater for nondrinking purposes.	Gardening, toilet flushing, clothes washing, and hot water systems.	[32]

Tasmania (TAS)	No governing policy in place.	A local council plumbing permit approval is required for tank installation. Works must be carried out by an accredited plumber.	Essentially outdoor use.	[10]

Northern Territory (NT)	The Building Code of Australia, National Plumbing Code (AS/NZS 2003b; DCC 2007). The plumbing guideline is governed by the Northern Territory Land Group (NTLG).	No mandatory requirement.	Toilet flushing, laundry use, gardening for outdoor use, firefighting, cooling tower, and cold water use.	[10]

Australia Capital Territory (ACT)	The AS/NZS 3500 Section 4 regulates the installation of rainwater tank on a residential property. Tanks must be installed at least 3 m from the rear boundary and 1.5 m from the side building boundary.	Tanks of less than 17 kL installed at 2.4 m above ground level do not need council approval. Larger tanks require approval from the ACT Planning and Land Authority (ACT-PLA) or building approval from a private certifier or both.	Toilet flushing, laundry use for indoor use and gardening, firefighting, and cooling tower for outdoor use.	[10]

**Table 2:** Requirements for rebates on rainwater tank systems.

State	Fund allocation	Reference
South Australia	Up to $1,000 granted by SA water to purchase tank and get them plumbed for nonpotable use. Program ended in March 2013	[10, 27]

Victoria	Rebates from $500 to $1,500. Program ended 30 June 2015	[48]

New South Wales	Up to $1,500 for tanks not installed under the BASIX regulation. Up to $500 offered by Sydney Water to schools to purchase tanks with an extra $500 to get them plumbed in for nonpotable use. Program ended 30 June 2009	[27, 49]

Queensland	Rebates up to $1500 for a 3000 L tank or larger if plumbed in for nonpotable use. Program ended 31 December 2008	[10, 27]

Western Australia	A rebate up to $600 for tanks larger than 2,000 L if plumbed in for nonpotable use. Program ended 30 June 2009	[10, 27]

Australian Capital Territory	From $750 to $1,000 for new tanks if plumbed in for nonpotable use. $600 to plumb in an existing tank. Program ended in 2008	[10]

Tasmania	In Hobart, up to $170 for outdoor use, $220 if plumbed in for nonpotable use for tanks of at least 600 L capacity. Program ended 30 June 2008	[10]

Northern Territory	No rebate scheme was granted	

**Table 3:** Trace metals found in rainwater in key Australian towns and cities (in ppm). ^*∗*^Aesthetic only for zinc and lead^*∗*^ (total lead).

Location	Metal concentration	Health limit	Times above the limit	Reference
Adelaide, SA	15.8 zinc	3^*∗*^	5.2 times higher	[50]

Port Pirie, SA	0.06 lead	0.01	0.6 times higher	[51]

Adelaide, SA	0.03 lead^*∗*^	0.01	3 times higher	[52]
	16.1 zinc	3^*∗*^	5.3 times higher	

Melbourne, VIC	0.42 lead	0.01	42 times higher	[53]
	0.1 chromium	0.05	2 times higher	
	0.17 nickel	0.02	8.5 times higher	

Melbourne, VIC	0.5 lead	0.01	50 times higher	[54]

Newcastle, NSW	0.02 cadmium	0.002	10 times higher	[55]
	0.14 arsenic	0.01	14 times higher	
	0.81 chromium	0.05	16 times higher	
	15 copper	2	7.5 times higher	

Newcastle, NSW	0.21 chromium	0.05	4.2 times higher	[56]

Sydney, NSW	0.55 arsenic	0.01	55 times higher	[51, 57]
	2.78 lead	0.01	278 times higher	
	0.33 lead	0.01	33 times higher	

Esperance, WA	0.01 lead	0.01	1.2 times higher	[58]
	0.03 nickel	0.02	1.5 times higher	

Karumba, QLD	0.006 cadmium	0.002	3 times higher	[59]
	0.10 lead	0.01	10 times higher	
	10.8 zinc	3^*∗*^	3.6 times higher	

Brisbane, QLD	0.85 lead	0.01	85 times higher	[60]
	0.03 arsenic	0.01	3 times higher	
	0.009 cadmium	0.002	4.5 times higher	
	26 zinc	3^*∗*^	9 times higher	

**Table 4:** Trace metals seasonal variability in rainwater harvested in Newcastle [56] (in ppm).

Parameters	Health limit	Site 1		Site 2	
		Winter	Summer	Winter	Summer
Silver	0.1	0.032	0.009	0.047	0.014
Cadmium	0.002	0.18	0.17	0.05	0.10
Lead	0.01	2.78	10.5	3.59	5.77
Uranium	0.017	0.003	0.003	0.002	0.002
Manganese	0.5	20.0	82.3	6.95	12.1
Chromium (Cr^6^)	0.05	0.09	0.21	0.03	0.05
Arsenic	0.01	0.25	0.55	0.08	0.09
Zinc	3^*∗*^	518	725	77.2	150
Copper	2	0.08	0.25	0.10	0.16
Nickel	0.02	0.29	0.16	1.47	2.72

^*∗*^Aesthetic only for zinc.

**Table 5:** Prevalence of organisms in rainwater collected in key Australian cities.

Location	Organisms	Occurrence (%)	Count (CFU/100 mL)	Reference
Adelaide, South Australia	*Legionella *spp.	17	840,000	[61]
	*E. coli*	42	250	
	*Salmonella *spp.	8	*∗*	
	Enterococci	67	450	
	*Aeromonas *	33	1700	

Brisbane, Queensland	*E. coli*	36	260	[61]
	*E. coli*	*∗*	2,420	[62]
	*C. perfringens*	100	55	[63]
	Enterococci	70	19	

Broken Hill, New South Wales	*Legionella *spp.	70	73,000	[61]
	Enterococci	70	37	
	*C. perfringens *	70	16	
	*Aeromonas *	10	22	

Canberra, Australian Capital Territory	*E. coli *	50	9,200	[61]
	Enterococci	100	32,000	
	*Campylobacter *spp.	10	43	
	*Legionella *spp.	10	20,000	

Newcastle, New South Wales	*Pseudomonas *spp.	60	15,200	[64]
	*E. coli*	*∗*	17	[65]

Southern Queensland, Queensland	*E. coli*	63	89	[66]
	*Campylobacter *spp.	60	50	
	Enterococci	92	91	
	*Salmonella *spp.	4	700	
	*Giardia lamblia*	30	580	

Sydney, New South Wales	Enterococci	100	199	[61]
	*E. coli*	100	3,900	
	*C. perfringens *	33	16	

Wollongong, New South Wales	*E. coli*	100	100	[61]
	Enterococci	92	30,000	
	*C. perfringens *	42	27	
	*Aeromonas *	33	*∗*	

^*∗*^No data available.

**Table 6:** Incidents of illness and diseases outbreak linked with drinking rainwater. ^*∗*^Declared outbreak.

Year	State	Microorganisms	Place	Incidents	Evidence	Reference
1981	New South Wales	*Clostridium botulinum*	Home location	3	High	[67]
1997	Queensland	*Campylobacter*	Nursing home	23^*∗*^	High	[68]
1999	Queensland	*Salmonella *spp.	Working camp	28	High	[69]
2001	Queensland	*Salmonella *spp.	Nursing home	3	High	[70]
2004	Queensland	*Salmonella *spp.	Nursing home	8	High	[70]
2004	Victoria	*Campylobacter*	Nursing home	7	Suspicion	[71]
2005	Queensland	*Salmonella *spp.	Nursing home	8	High	[71]
2005	New South Wales	*Giardia lamblia*	Not specified	*∗*	High	[72]
2006	Queensland	*Campylobacter*	Holiday camp	46	High	[71]
2006	South Australia	*Cryptosporidiosis*	Home location	19	High	[73]
2007	Victoria	*Salmonella*	School camp	27^*∗*^	High	[74]
2009	Queensland	*Campylobacter*	Island resort	29	High	[73]
